# Opium

**Published:** 1888-08

**Authors:** J. D. Tyrrell


### OPIUM.

Mr. President and Gentlemen:—To give an exhaustive paper on Opium in convulsions would be to make my paper too long, so I shall merely give a few leading symptoms. While it will not do to state about any remedy that it will not act save in such a class of complaints, I think one can safely say that Opium is more likely to be indicated in *recent rather than chronic conditions*, and in diseases occurring in either extreme of life, the very young or the aged.

The face is usually very dark red; the darker red the face the more Opium will be indicated; veins distended; face is not only red, but looks inflamed, and the lips are swollen; eyes look wild, red, and protrude from their sockets. Tetanus, the back is stiff and straight; opisthotonus, the trunk is curved in the form of an arch.

Apoplexia, with vertigo, buzzing in ears, unconscious, red, bloated face, red, half-closed eyes, dilated, insensible pupils, foam at mouth, convulsive movements of limbs, and *slow, stertorous*

breathing, which *stertor is continuous.* Puerperal convulsions during and after labor, with unconsciousness and drowsiness, or coma between the paroxysms.

Epilepsy, from fear or fright, or after bitter reproaches; epileptic fits, *especially at night or toward morning*, with loss of consciousness (conscious, Hell.), violent movements of limbs, and suffocative paroxysms.

Opium has apoplexy, with half-closed eyes; heat, with sweat; paralysis of the *right side of body.*

Aconite has apoplexy, with staring eyes, hot head, cold extremities, and paralysis of *left side of the body.*

Camphora has predominantly apoplexia nervosa, no paralysis of limbs; Opium, apoplexia sanguinea, paralysis of limbs.

Opium, apoplexia sanguinea, *eyes protruding;* Ant. tart., apoplexia nervosa, and Serosa, *eyes sunken.*

Opium has apoplexia more frequent than paralysis; the *paralysis is painless;* jerks, the flexor muscles only active; screams with convulsions, loss of consciousness; Plumbum has paralysis more frequent than apoplexy; the *paralysis* is predominantly painful; jerks, flexor and extensor muscles alternately; rarely screams with convulsions; conscious. Puerperal convulsions of Stram. have copious sweating.

Predominant effects of Opium: general *insensibility* of *the whole nervous system; diseases of drunkards;* of *senile age; tremor* of whole body, *with jerking and twitching of limbs; convulsions and spasms*, epilepsy, tetanus, opisthotonus, apoplexia; *bad consequences of fright* if Opium be resorted to *immediately* after fright; if used too late Opium does mischief.

J. D. TYRRELL.

